# INP1 involvement in pollen aperture formation is evolutionarily conserved and may require species-specific partners

**DOI:** 10.1093/jxb/erx407

**Published:** 2017-11-28

**Authors:** Peng Li, Samira Ben-Menni Schuler, Sarah H Reeder, Rui Wang, Víctor N Suárez Santiago, Anna A Dobritsa

**Affiliations:** 1 Department of Molecular Genetics and Center for Applied Plant Science, Ohio State University, Columbus, OH 43210, USA; 2 Department of Botany, University of Granada, 18071 Granada, Spain

**Keywords:** Arabidopsis, evolutionary analysis, exine, INP1, maize, membrane domains, plant reproduction, pollen aperture, pollen germination

## Abstract

Pollen wall exine is usually deposited non-uniformly on the pollen surface, with areas of low exine deposition corresponding to pollen apertures. Little is known about how apertures form, with the novel Arabidopsis INP1 (INAPERTURATE POLLEN1) protein currently being the only identified aperture factor. In developing pollen, INP1 localizes to three plasma membrane domains and underlies formation of three apertures. Although INP1 homologs are found across angiosperms, they lack strong sequence conservation. Thus, it has been unclear whether they also act as aperture factors and whether their sequence divergence contributes to interspecies differences in aperture patterns. To explore the functional conservation of INP1 homologs, we used mutant analysis in maize and tested whether homologs from several other species could function in Arabidopsis. Our data suggest that the INP1 involvement in aperture formation is evolutionarily conserved, despite the significant divergence of INP1 sequences and aperture patterns, but that additional species-specific factors are likely to be required to guide INP1 and to provide information for aperture patterning. To determine the regions in INP1 necessary for its localization and function, we used fragment fusions, domain swaps, and interspecific protein chimeras. We demonstrate that the central portion of the protein is particularly important for mediating the species-specific functionality.

## Teaser

The role of INP1as a pollen aperture factor seems to be evolutionarily conserved but may need species-specific partners. The central portion of INP1 is particularly important for species-specific functionality.

## Introduction

Deposition of pollen wall exine leads to the formation of beautiful geometrical patterns on the surfaces of pollen grains (Kesseler and Harley, 2004). A very common type of pollen patterning elements are apertures, the regions on the pollen surface where exine deposition is absent or reduced. Aperture patterns, defined by aperture number, positions, and morphology, are usually highly stereotypical within pollen grains of the same plant species, but vary widely across the species of angiosperms (Wodehouse, 1935; Furness and Rudall, 2004; www.paldat.org).

Stereotypical aperture development indicates that the pollen surface has polarity, and that apertures develop at distinct domains that must be specified differently from the rest of the pollen surface. This, combined with the enormous diversity of pollen aperture patterns across plant species, makes apertures a unique model of cellular and extracellular polarity. Although the processes of polarity generation and aperture formation in developing pollen have been drawing attention for a long time (Wodehouse, 1935; Heslop-Harrison, 1963, 1968, 1971; Skvarla and Larson, 1966; Dover, 1972; Sheldon and Dickinson, 1983, 1986, Ressayre *et al.*, 1998, 2003; Albert *et al.*, 2010; Reeder *et al.*, 2016), the details of the mechanism that specifies apertures and restricts exine deposition at the distinct sites on the pollen surface have remained elusive.

The first signs of apertures become apparent after the male meiotic cytokinesis, when the products of meiosis—the sister microspores which will develop into four pollen grains—are transiently kept together as a tetrad by the common callose wall (Heslop-Harrison, 1971; Albert *et al.*, 2010; Dobritsa and Coerper, 2012). The close temporal association between meiosis and aperture development, as well as the spatial correlation in many species between the positions of apertures and the positions of the cell plate closures at the end of meiotic cytokinesis, has led to the hypotheses that meiosis and/or cell partitioning by cytokinesis may provide positional clues for aperture formation (Wodehouse, 1935; Heslop-Harrison, 1971; Dover, 1972; Sheldon and Dickinson, 1983, 1986, Ressayre *et al.*, 1998, 2002; Albert *et al.*, 2010), although the nature of these clues is unknown. Additionally, the tight contacts between the membrane domains at the future aperture sites and the overlying regions of callose wall appear to be important for aperture development, as they are likely to serve to limit deposition of the exine precursor, the primexine, at these positions and, therefore, drive formation of apertures (Dobritsa *et al.*, 2017).

In the wild-type pollen grains of Arabidopsis (*Arabidopsis thaliana*), apertures form as three equidistant longitudinal furrows (Bronkers, 1963; Dobritsa *et al.*, 2011). This pattern suggests that the three equidistant domains on the surface of developing pollen grains where exine is not deposited must have a different molecular composition from that of the nearby regions where exine is deposited in a uniform reticulate pattern. We previously discovered one molecular player that contributes to the generation of aperture domains and to pollen aperture formation in Arabidopsis (Dobritsa and Coerper, 2012; Reeder *et al.*, 2016). INAPERTURATE POLLEN1 (INP1) is a novel plant-specific protein with no recognizable domains of known function, which acts as an essential aperture factor. Pollen of the *inp1* null mutants completely lacks apertures (Dobritsa *et al.*, 2011; Dobritsa and Coerper, 2012). INP1 pre-marks positions of apertures in developing microspores by specifically localizing to three equidistant membrane domains at the surface of tetrad-stage microspores and assembling at these sites into three punctate lines (Dobritsa and Coerper, 2012; Reeder *et al.*, 2016). Such a distinct and unusual pattern of protein localization suggests the existence of molecular mechanisms that help specify three narrow plasma membrane regions as future aperture sites and guide INP1 to these positions at the membrane. However, the absence of domains of known function in INP1 makes it difficult to predict how it localizes to specific membrane areas and contributes to aperture formation.

Arabidopsis pollen with its three equatorial furrow-shaped apertures (tricolpate) exhibits the prototypical and the most common aperture pattern in eudicots, although many variations in aperture patterns and morphology exist within the eudicot clade (Wodehouse, 1935; Furness and Rudall, 2004; www.paldat.org). In contrast, pollen of monocots usually has very different aperture patterns, most commonly developing a single aperture in the shape of a furrow (monosulcate) or a pore (ulcerate) (Wodehouse, 1935; Zavada, 1983; Linder and Ferguson, 1985; Ressayre *et al.*, 2002; Furness and Rudall, 2004; www.paldat.org).

**Fig. 1.**
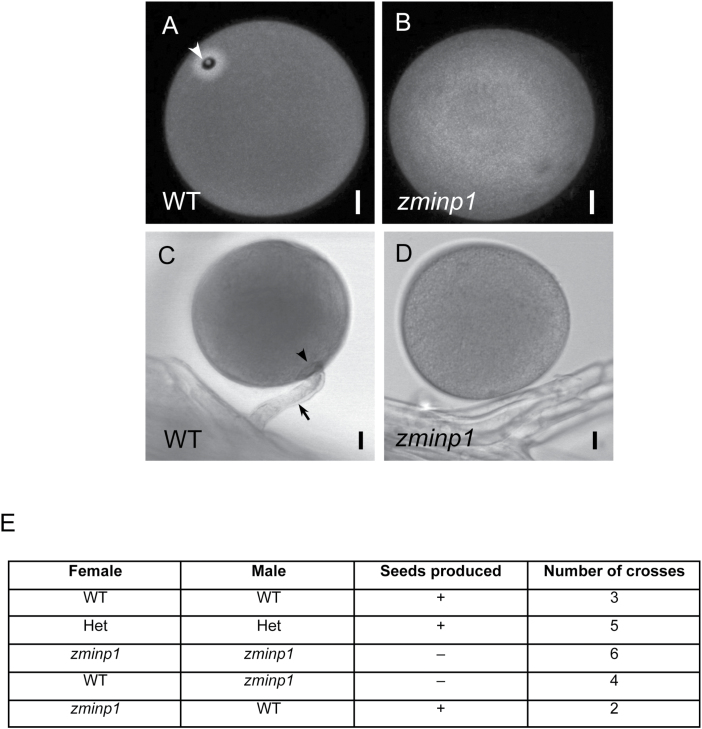
INP1 ortholog from maize is involved in formation of a single pore-like aperture, which is required for pollen tube germination. (A, B) Auramine O-stained exine of the wild-type (A) and *zminp1* mutant (B) pollen. The arrowhead in (A) points at the aperture surrounded by a brightly stained annulus. A lid-like operculum is visible as a dot inside the aperture. None of the *zminp1* pollen grains had apertures. (C, D) Wild-type (C) and *zminp1* (D) pollen grains after 24 h of on-silk germination. The arrowhead in (C) points to the aperture and the arrow points to the pollen tube. While many of the wild-type pollen grains had pollen tubes associated with them (72%, *n*=160), none of the *zminp1* pollen germinated pollen tubes (*n*=247). Scale bars=10 μm. (E) Results of crosses between plants with the indicated phenotypes: WT, homozygous wild type; Het, a heterozygote for *zminp1* mutation; *zminp1*, a homozygous mutant.

Although putative homologs of INP1 have been identified in most angiosperms with available genomic or transcriptomic data, their sequences are not strongly conserved across plant species (Dobritsa and Coerper, 2012). In particular, in grasses (Poaceae), INP1 proteins have diverged very significantly from their eudicot counterparts (<40% sequence identity). Within the Poaceae family, however, sequences of INP1 homologs are highly conserved—exhibiting 87–95% protein identity in pairwise comparisons between INP1s from maize (*Zea mays*), rice (*Oryza sativa*), Brachypodium (*Brachypodium distachyon*), *Setaria italica*, and *Sorghum bicolor* (Dobritsa and Coerper, 2012). Interestingly, aperture patterns are also highly similar between different grasses and distinctly different from the eudicot patterns (Wodehouse, 1935; Linder and Ferguson, 1985; www.paldat.org): apertures in grasses are represented by a single germinal pore that occupies a small portion of the pollen surface (Fig. 1A).

Previously, we hypothesized that differences in INP1 sequences could be responsible for differences in aperture morphology in different species. In our previous study, we set out to test this hypothesis by expressing a homolog from the grass Brachypodium (BdINP1) in the developing wild-type and *inp1* pollen of Arabidopsis and testing whether it could affect or restore formation of apertures. We found, however, that expression of BdINP1 in Arabidopsis had no influence on aperture development (Dobritsa and Coerper, 2012), suggesting either that BdINP1 needs additional partners that are present in Brachypodium and absent from Arabidopsis or that homologs of INP1 in grasses are not involved in formation of apertures.

In the current study, we had two main objectives: (i) to test the evolutionary preservation of the INP1 function; and (ii) to identify regions in the INP1 protein that are necessary for its localization and function. With the first objective in mind, we identified a mutant in which a close homolog of BdINP1 from another grass—maize (ZmINP1)—is disrupted, and found that pollen of this mutant lacks apertures, indicating that despite the significant changes observed both in the INP1 protein sequences and in the aperture patterns of grasses compared with eudicots, the involvement of INP1 proteins in pollen aperture formation has been preserved in evolution. To look further at the INP1 functional conservation, we have tested the ability of less divergent INP1 homologs from several eudicot families to function in Arabidopsis. We demonstrate that although homologs from members of the Brassicaceae family could substitute for the loss of AtINP1, homologs from Solanaceae and Papaveraceae were not functional in Arabidopsis. This finding is consistent with the model predicting that additional aperture factors are required to act in conjunction with INP1 and that these factors are divergent across species. Finally, to identify regions in the INP1 protein that are necessary for its function and unique localization, we performed a structure–function analysis of INP1 and tested the ability of a series of protein fragments, domain-swapped constructs, and interspecific chimeras to restore apertures and form punctate lines in Arabidopsis. We found that the ability of INP1 to function and localize correctly required almost the entire protein sequence. However, the central portion of the protein was particularly important for mediating the species-specific functionality of INP1.

## Materials and methods

### Plant material and growth conditions

All plants, except for maize, were grown at 22 °C with a 16 h light:8 h dark cycle in the growth chambers or in the greenhouse at the Biotechnology facility at Ohio State Univesity (OSU). In addition to Arabidopsis (*inp1-1*, Columbia background), DNA and/or tissues of the following species were used: *Capsella rubella* (CS22561), *Matthiola incana* [common name—stock; seeds obtained from a web-based gardening center (sarahraven.com)], tomato *Solanum lycopersicum* (Heinz 1706), *Brachypodium dystachion* (Bd21), and *Eschscholzia californica* (GDA 52801). The UFMu-02338 maize transposon insertion line was obtained from the Maize Genetics Cooperation Stock Center. Maize plants from the UFMu-02338 line, along with the background line W22, were grown at 24–29 °C with a 16 h light:8 h dark cycle in the greenhouse at the Biotechnology facility or under ambient summer conditions in the field at the Waterman Farm Research Facility at OSU.

### Maize fertility and pollen germination assays

Field-grown plants were used to assess maize fertility and pollen germination ability. The genotypes of the plants were established using two sets of primers (Supplementary Table S1 at *JXB* online): ZmINP1-BF and ZmINP1-BR primers amplify the wild-type band, and TIR6 and ZmINP1-BR primers detect the presence of the UniformMu transposon in *ZmINP1*. All ears were bagged before silk emergence. Silks that started emerging were cut and pollination with freshly shed pollen was performed after they grew back to ~2 cm. To assay pollen germination, pollinated silks were harvested 24 h after pollination, and pollen tube presence was assessed using a Nikon A1+ confocal microscope with a ×40 oil-immersion objective. To determine the seed siring ability, pollinated ears were kept for ~45 more days, and then collected and dried.

### Confocal microscopy

Confocal microscopy of mature pollen grains and of tetrads of microspores was performed, as described in Reeder *et al.* (2016), using a Nikon A1+ confocal microscope with a ×100 oil-immersion objective (NA=1.4). Exine of mature pollen stained with auramine O was excited with a 488 nm laser and emitted fluorescence was collected at 500–550 nm. In tetrads, yellow fluorescent protein (YFP) was excited with a 514 nm laser line and fluorescence emission was collected at 522–555 nm; Calcofluor White-stained callose walls were excited with a 405 nm laser line and their fluorescence was collected at 424–475 nm.

### Transgenic constructs

The primers used to create all constructs are listed in Supplementary Table S1. The *DMC1pr:INP1-YFP-pGR111* full-length construct was used as a basis for the constructs generated in this study. To create fragments of INP1 fused with YFP, the *DMC1pr:INP1-YFP* construct was digested with *Age*I and *Nco*I, and the full-length *INP1* was replaced by the truncated versions that were inserted between the *DMC1* promoter (*DMC1pr*) and *YFP*. *AtINP1* was similarly replaced with sequences of *INP1* homologs from other species. A genomic intron-containing fragment was used for *EcINP1*. *INP1* homologs from other species were either intronless or, in the case of *SlINP1*, the short intron was removed during cloning by using a forward primer that contained the short first exon at its 5' end and the beginning of the second exon at its 3' end.

To create most of the constructs for the experiments on putative transmembrane (TM) regions, the regions corresponding to the full-length INP1, INP1ΔC, C-terminal regions from INP1 homologs of other species, or the FERONIA (FER) TM were PCR amplified with the respective primers (Supplementary Table S1). In each case, a corresponding combination of two fragments was cloned between the *DMC1pr* and *YFP* in the *Age*I–*Nco*I-digested vector using the In-Fusion technology (Clontech). The In-Fusion-based strategy was also used to combine the Arabidopsis and tomato INP1 fragments to create the interspecific chimeras. To create the *DMC1pr:INP1-YFP-FER TM* construct, *INP1-YFP* without the stop codon was amplified using *DMC1pr:INP1-YFP* as a template, along with *FER TM*, and the two fragments were cloned using In-Fusion into the *Age*I–*Spe*I-digested vector. We used the same FER region as in the previous Liu *et al.* (2016) study: the region contained the 24 amino acid FER TM sequence flanked by four amino acids at the N-terminus and nine amino acids at the C-terminus (Liu *et al.*, 2016).

High-fidelity DNA polymerases Phusion (New England Biolabs) or Clone-Amp Hi-Fi (Clontech) were used for all PCR amplifications. All constructs were verified by sequencing prior to transformation into the Agrobacterium strain GV3101. *inp1-1* plants were transformed by the floral dip method (Clough and Bent, 1998); transgenic plants were selected with BASTA, and the presence of transgenes was confirmed with specific primers. A minimum of 10 T_1_ plants per construct were examined for phenotypes.

### Identification of *MiINP1*

To identify the *INP* homolog from *M. incana*, we used a combination of genomic DNA amplification and 5'- and 3'-RACE experiments on transcripts isolated from young buds. Initially, forward and reverse primers (Min-4F and Min-5R, Supplementary Table S1) were designed based on the consensus information from the available sequences of *INP1* homologs from multiple Brassicaceae and used to amplify Matthiola genomic DNA. The PCR product was sequenced and found to be homologous to *AtINP1*. To identify the sequences of the 5' and 3' ends of the gene, 5'- and 3'-RACE experiments were performed using the First-Choice RLM-RACE kit (Ambion) according to the manufacturer’s instructions. For template, RNA was isolated from young buds of *M. incana*, and cDNA was created as described (Dobritsa and Coerper, 2012). Based on the RACE results, the new F and R primers (Min-EF and MiINP1-14R, Supplementary Table S1) were then designed and used to amplify the full-length MiINP1 ORF.

### Identification of *EcINP1*

BLAST searches were performed with the INP1-like sequence from another basal eudicot, *Aquilegia coerulea* (Aquca_013_00700), against the transcriptomic sequences of *E. californica* obtained by the 1000 Plants Project (Wickett *et al.*, 2014). One of the identified sequences (scaffold ERXG-2062521) included an *INP1*-like coding sequence (CDS) that was used to retrieve additional *E. californica* scaffolds (TUHA-2055946, UNPT-2055332, and EVOD-2009760) also containing *INP1*-like sequences. Notably, the TUHA-2055946 transcript was obtained from a flower bud sample, suggesting that EcINP1 is expressed at the right places and developmental stages for being an aperture factor. The alignment of the resulting sequences allowed us to predict the putative full-length version of the *EcINP1* CDS. Primers (EcaINP1-F and -R, Supplementary Table S1) were then designed to amplify the *EcINP1* gene from genomic DNA.

### Accession numbers

The following identifiers are used for the *INP1* homologs used in this study: *AtINP1* (At4g22600, Arabidopsis Genome Initiative), *BdINP1* (XM_003569989, GenBank/EMBL), *CrINP1* (Carubv10006857m, Phytozome), *EcINP1* (LT840341), *MiINP1* (KY829106, GenBank/EMBL), *SlINP1* (Solyc08g079050, Sol Genomics Network), and *ZmINP1* (GRMZM2g112914, MaizeGDB).

## Results

### The function of INP1 as a pollen aperture factor is conserved between Arabidopsis and maize, despite the divergence of protein sequences and aperture morphologies

Previously, we demonstrated that the *INP1* homolog from the grass *B. distachyon* (*BdINP1*) was unable to restore pollen apertures in the Arabidopsis *inp1* mutant (Dobritsa and Coerper, 2012). To test whether the INP1 proteins from grasses that have significantly diverged from the eudicot INP1 proteins are involved in aperture formation, we obtained a transposon insertion (UFMu-02338) from the maize UniformMu population (McCarty *et al.*, 2005). The transposon was inserted into the middle of the ORF of the maize homolog of INP1 [*ZmINP1* (*GRMZM2g112914*)]. Homozygous *zminp1* mutants produced pollen which, like the *inp1* pollen in Arabidopsis, completely lacked apertures but had otherwise normally formed exine and normal pollen morphology (Fig. 1A, B). This finding demonstrates that despite the very significant differences between grasses and eudicots in both the structures of apertures and the sequences of INP1 protein, the role of INP1 as a specific pollen aperture factor is nevertheless conserved.

In the past, we showed that in Arabidopsis the loss of apertures is well tolerated by pollen and does not have a strong negative impact on its fertility (Dobritsa *et al.*, 2011). The discovery of the inaperturate mutant in maize allowed us to assess the importance of single germinal pores for plant fertility in grasses. We found that the requirement for the presence of apertures in maize is much more stringent than in Arabidopsis, as inaperturate maize pollen completely lost its ability to set seeds and grow pollen tubes (*n*=247) in the *in vivo* assays (Fig. 1D, E). A recent study found that in Arabidopsis, even in the case of wild-type pollen grains, pollen tubes often emerge through the exine wall and not through the apertures (Edlund *et al.*, 2016). In contrast, in the wild-type maize, we never observed pollen tubes emerging outside of the aperture region (*n*=115) (Fig. 1C). Taken together, these results indicate that, in maize, apertures are a critical factor for pollen fertility and, therefore, for plant fitness.

### Although INP1 orthologs from the Brassicaceae are functional in Arabidopsis, INP1 proteins from more distant eudicot species are unable to function in Arabidopsis

Compared with INP1s from grasses, orthologs from eudicot species are more closely related to the Arabidopsis INP1 (AtINP1) and, in general, exhibit sequence similarity consistent with the evolutionary relationships between the species (Dobritsa and Coerper, 2012). To test if eudicot orthologs could substitute for the AtINP1 function, we created a series of constructs containing INP1s from the following families, clades, and species: Brassicaceae (rosids), *C. rubella* (*CrINP1*) and *M. incana* (*MiINP1*); Solanaceae (asterids), tomato (*S. lycopersicum*, *SlINP1*); and Papaveraceae (basal eudicots), California poppy (*E. californica*, *EcINP1*). CrINP1 and MiINP1 are from the species that belong to the same family as Arabidopsis, and these proteins are closely related to AtINP1 (92% and 79% amino acid identity, respectively), whereas SlINP1 and EcINP1 have diverged more significantly from AtINP1 (47% and 44% amino acid identity, respectively). Analysis of tomato transcriptomics data available through the Tomato Functional Genomics Database showed that, like AtINP1, SlINP1 is predominantly expressed in young flower buds, consistent with its involvement in pollen development. In addition, the *EcINP1* transcript is also present in flower bud samples generated by the 1000 Plants project (www.onekp.com).

**Fig. 2.**
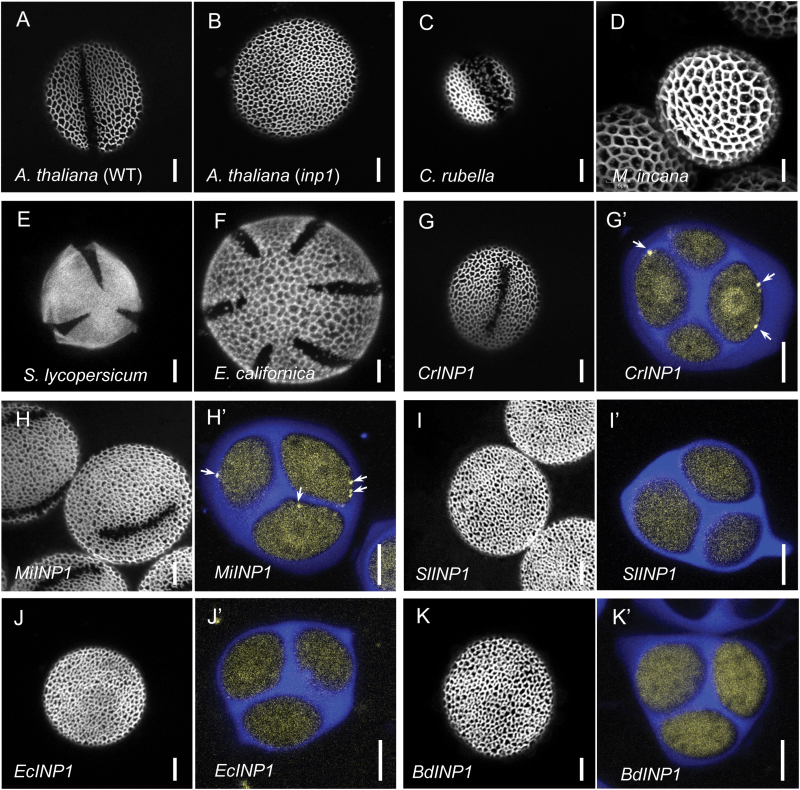
INP1 orthologs from *Brassicaceae* species can substitute for AtINP1, while orthologs from more distant families fail to do so. (A–D) Pollen from the eudicot species used in this study. (A) Pollen of wild-type *A. thaliana*. One aperture is visible in this view. (B) Pollen of the *inp1* mutant of *A. thaliana* completely lacks apertures. (C) Pollen in *C. rubella* has apertures that are wider than in Arabidopsis and have irregular margins and internal sporopollenin deposits (a portion of the pollen surface with an aperture is visible). (D) Pollen in *M. incana* lacks apertures. (E) Pollen from tomato *S. lycopersicum* has three colporate apertures (polar view). (F) Pollen from California poppy, *E. californica*, often has six colpate apertures (polar view). (G–K') Pollen aperture phenotypes (G, H, I, etc.) and INP1–YFP fluorescence in tetrads (G', H', I', etc.) from the Arabidopsis *inp1* plants transformed with constructs containing YFP-fused INP1 homologs from *Capsella rubella* (G, G'), *Matthiola incana* (H, H'), *Solanum lycopersicum* (I, I'), *Eschscholszia californica* (J, J'), and *Brachypodium dystachyon* (K, K'). Callose wall of tetrads is stained with Calcofluor White (blue). Yellow signal indicates the presence of INP1–YFP. Arrows point to the INP1–YFP puncta. Scale bars=5 μm.

It is noteworthy that pollen of *M. incana* lacks apertures (Furness, 2007; Fig. 2D). Part of the reason for including MiINP1 in our study was to determine whether the aperture defects in Matthiola could be attributed to the loss of INP1 function. Pollen from all other eudicot species used here, similar to Arabidopsis (Fig. 2A), has furrow-like apertures, albeit with some variations in morphology or number (www.paldat.org;Fig. 2C, E, F).

To create complementation constructs, the genes of the *INP1* homologs were placed under the control of the *DMC1* promoter (Klimyuk and Jones, 1997), which was shown to provide strong expression of AtINP1–YFP at the tetrad stage and to ensure the robust complementation of aperture defects in the *inp1* mutant (Reeder *et al.*, 2016), with 100% of T_1_ plants (*n*=28) exhibiting aperture formation. The *INP1* homologs were fused with the *YFP* gene at their C-termini and transformed into the Arabidopsis *inp1* mutant. We then tested the ability of the resulting proteins to complement aperture defects in Arabidopsis and to assemble into the punctate lines at the periphery of the tetrad-stage microspores. In addition, to determine the subcellular localization in Arabidopsis of the Brachypodium INP1 (BdINP1), which was untagged in our previous study, we also created and transformed the *DMC1pr:BdINP1-YFP* construct.

Both CrINP1 and, interestingly, MiINP1 proteins successfully restored apertures in Arabidopsis pollen and formed punctate lines at the microspore periphery (Fig. 2G–H'). In contrast, the more divergent SlINP1 and EcINP1 failed both in restoring apertures and in forming lines in Arabidopsis, instead producing only diffuse YFP fluorescence (Fig. 2I–J'). Consistent with the previous BdINP1 results (Dobritsa and Coerper, 2012), BdINP1–YFP did not restore apertures, and the protein produced only diffuse fluorescence in microspores (Fig. 2K, K'). Notably, the apertures that were restored in the presence of CrINP1 had Arabidopsis-like morphology (Fig. 2G), which is different from wider apertures with irregular margins and internal exine deposits found in Capsella pollen (www.paldat.org;Fig. 2C). These results suggest that INP1 functionality has certain species specificity and that, by itself, INP1 does not control every aspect of aperture morphology.

### Only the very end of the INP1 C-terminus is dispensable for its localization and function

**Fig. 3.**
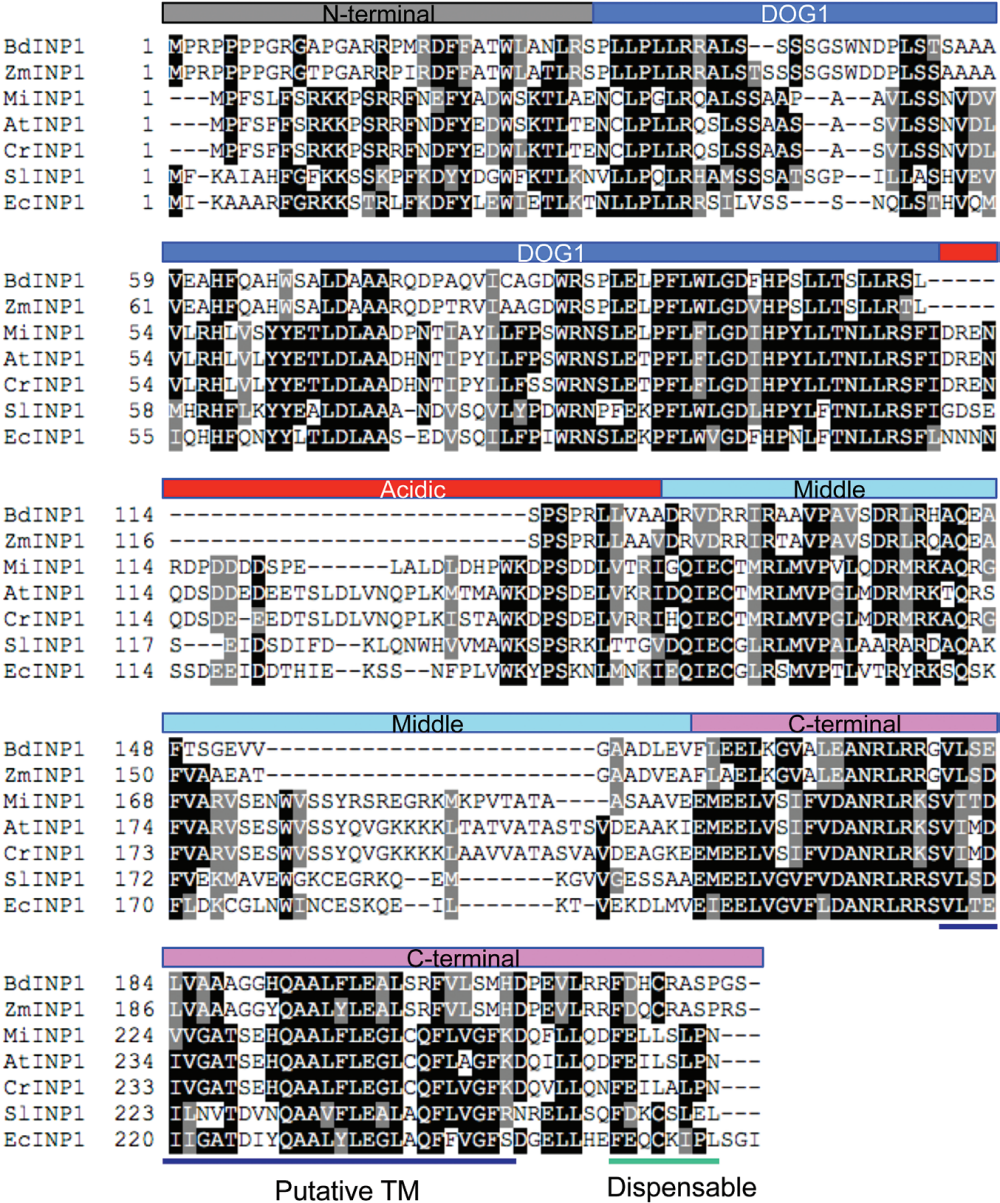
Alignment of the Arabidopsis INP1 (AtINP1) with its homologs from the species used in this study. Here AtINP1 was subdivided into five domains indicated above the alignment. Positions of the putative TM region and of the C-terminal amino acids that are dispensable for the function of AtINP1 are indicated below the alignment.

**Fig. 4.**
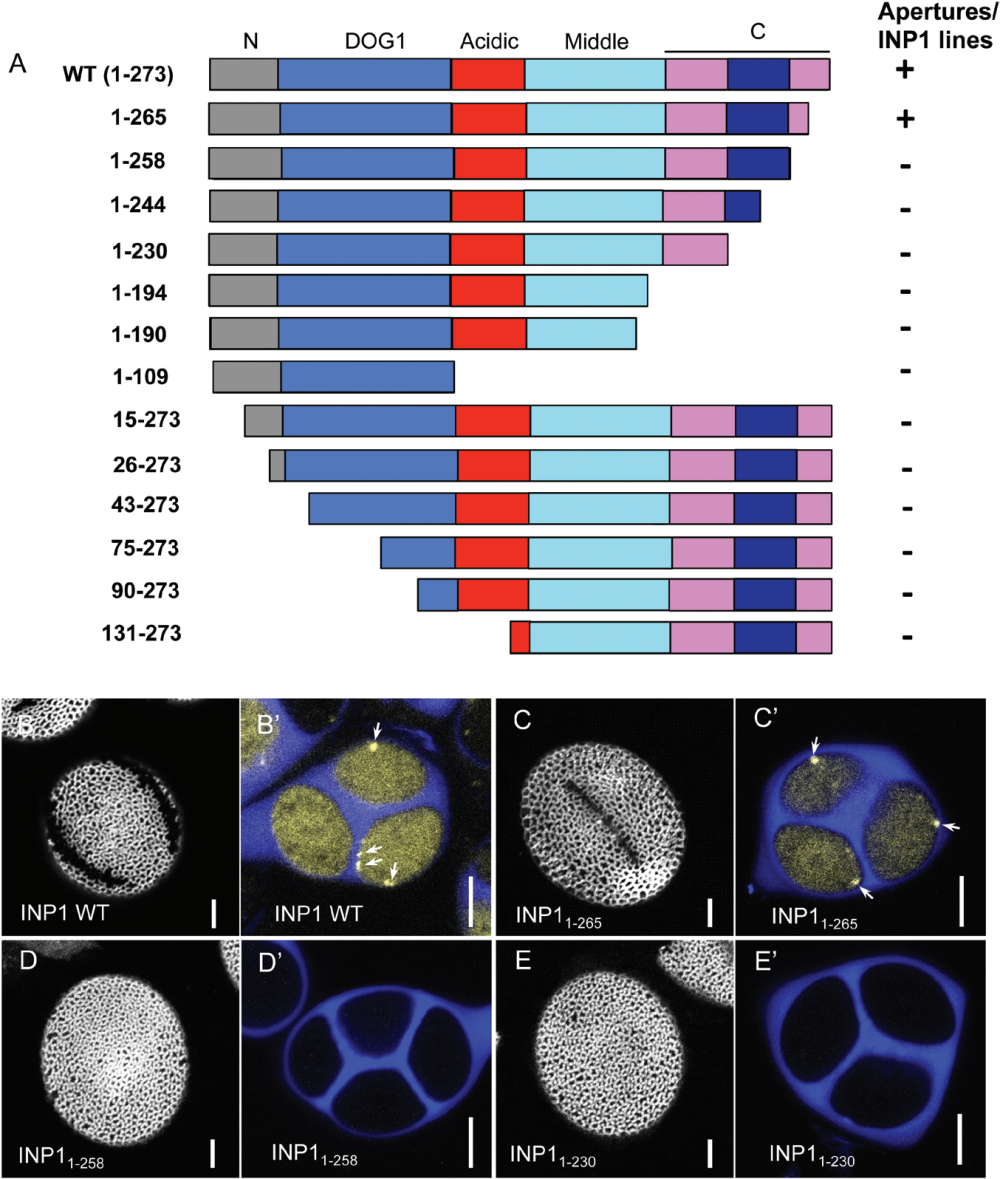
Only the very C-terminus of INP1 is dispensable for its localization and function. (A) A diagram of AtINP1 deletions with protein regions indicated and a summary of the ability of these truncated proteins to induce formation of INP1–YFP lines and restore apertures. The color scheme for protein domains is the same as in Fig. 3. The navy box indicates the putative TM domain. (B–E') Pollen aperture phenotypes (B, C, D, E) and YFP expression in tetrads (B', C', D', E') from lines transformed with these constructs. Shown are the examples from lines expressing the two constructs that rescued the aperture defects [wild type (B, B') and 1–265 (C, C')] and two constructs that did not rescue and lacked even diffuse YFP fluorescence [1–258 (D, D') and 1–230 (E, E')]. The callose wall of tetrads is stained with Calcofluor White (blue). Yellow signal indicates the presence of INP1–YFP. Arrows point to the INP1–YFP puncta. Scale bars=5 μm.

The unique localization of INP1 prompted us to ask which regions of the protein are required for its ability to localize to specific sites at the plasma membrane and to assemble into three lines. With the exception of the DOG1 domain of unknown function, INP1 lacks a clear domain organization (Dobritsa and Coerper, 2012). Still, after aligning it with homologs from other species, we can roughly divide AtINP1 into five regions (Fig. 3): the N-terminal domain (amino acids 1–30), the DOG1 domain (amino acids 31–109), the very divergent acidic domain (amino acids 110–149), the middle domain (amino acids 150–211), and the C-terminal domain (amino acids 212–273). Also, as noted previously (Dobritsa and Coerper, 2012), aligning AtINP1 with homologs from other plants helps to pinpoint several regions of higher evolutionary conservation, which could potentially fold into α-helixes, as well as more divergent regions that are expected to be structurally disordered. We used such structural predictions as an initial guide to create a series of constructs in which different portions of AtINP1 were tagged with YFP at their C-termini (Fig. 4A). The resulting constructs were placed under the control of the *DMC1* promoter, which allows the full-length construct to rescue robustly the aperture defects in the *inp1* mutant (Fig. 4B). We transformed these *INP1* fragment*–YFP* constructs into *inp1* and assessed pollen aperture formation and YFP signal localization in tetrads in the presence of the truncated proteins.

We found that only the non-conserved eight amino acid region at the very C-terminus was dispensable for the formation of the punctate INP1 lines and apertures (construct INP1_1–265_–YFP; Fig. 4A, C, C'). In contrast, all other constructs did not restore apertures or allow the punctate lines to form, and the tetrads expressing them lacked even the diffuse YFP fluorescence (Fig. 4A; representative images are shown in Fig. 4D–E'). This suggests that most of the INP1 protein is essential for its function and stability, and that it probably becomes destabilized when its portions are deleted. In parallel with these experiments, we also created a *DMC1pr:mRuby2-INP1* construct in which the full-length INP1 was fused with a fluorescent protein at the N-terminus. When transformed into the *inp1* mutant, this construct also did not restore aperture formation (Supplementary Fig. S1), suggesting that unlike fusions at the C-terminus, the presence of a tag at the N-terminus of INP1 interferes with the protein’s function.

### Testing the role of a putative transmembrane domain in INP1 localization

How INP1 is kept at the distinct plasma membrane regions that will become the sites of aperture formation is not known. Even though INP1 lacks clear domain organization and does not have recognizable signal peptides, some TM domain-predicting algorithms picked up a region at the C-termini in the INP1 homologs from multiple eudicot and monocot species as a possible TM domain (Dobritsa and Coerper, 2012). While these programs did not predict the existence of a TM domain in the INP1 proteins from Arabidopsis and other Brassicaceae, the significant similarity between this region in the Brassicaseae and in the species in which the TM domain was predicted (Dobritsa and Coerper, 2012) prompted us to explore this region more closely.

**Fig. 5.**
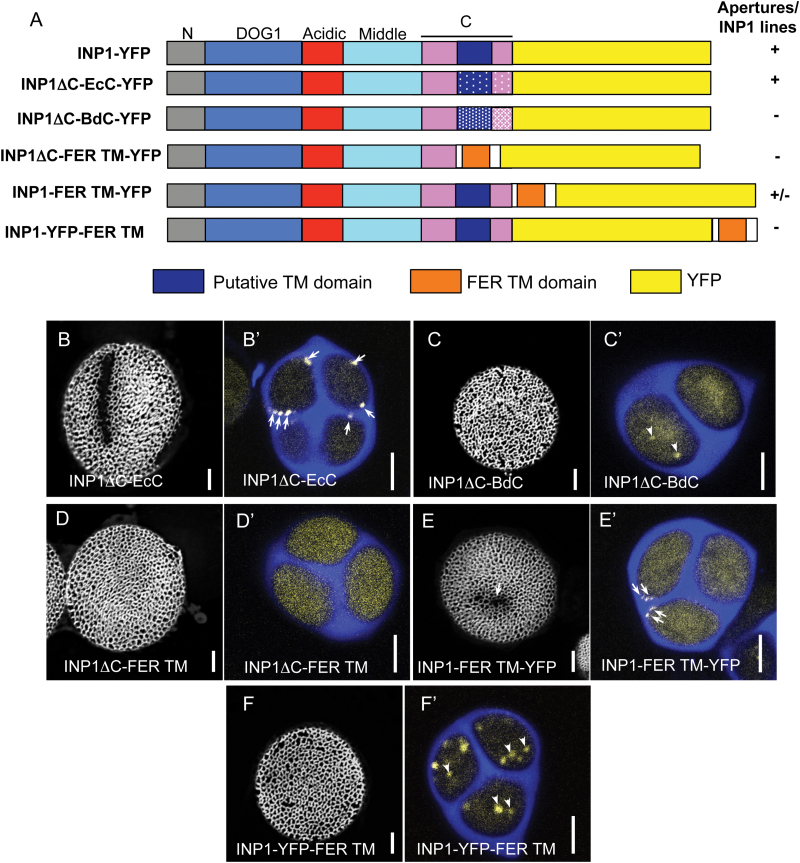
Delivery of INP1 to the microspore surface is required for aperture formation. (A) A diagram of INP1 constructs with the modified C-terminal regions, and a summary of the ability of these chimeric proteins to induce formation of INP1–YFP lines and restore apertures. Substitutions in the C-terminal domain by the corresponding regions from other species are indicated by stipple effects. The orange box indicates the TM domain from FERONIA and the white boxes surrounding it indicate several FER amino acids. The rest of the color scheme is the same as in Figs 3 and 4A. (B–F') Pollen aperture phenotypes (B, C, D, etc.) and INP1–YFP expression in tetrads (B', C', D', etc.) in lines expressing different constructs. The callose wall of tetrads is stained with Calcofluor White (blue). Yellow signal indicates the presence of INP1–YFP. INP1–YFP peripheral puncta and lines (arrows) were only visible in tetrads from plants expressing INP1ΔC–EcC (B') and INP1–FER TM (E') constructs. Short apertures (arrow) were produced in multiple INP1–FER TM T_1_ plants (E). Cytoplasmic puncta (arrowheads) were observed in tetrads expressing INP1ΔC–BdC (C') and INP1–YFP–FER TM (F'), suggesting that these modifications interfered with the ability of the protein to be transported to the cell periphery. Scale bars=5 μm.

In order to evaluate the importance and functional conservation of this region, as well as determine the consequences of having a *bona fide* TM domain added to AtINP1, we created five additional *DMC1pr*-driven constructs, in which the putative TM region was modified in some way. Each of these constructs was tagged with YFP at or near the C-terminus (Fig. 5A).

In three constructs, the C-terminus of AtINP1 (which included the putative TM domain as well as a short region immediately after, shown to be mostly dispensable for the AtINP1 function), was replaced by the following sequences: (i) a corresponding region from EcINP1 (EcC; construct INP1ΔC–EcC–YFP); (ii) a corresponding region from BdINP1 (BdC; construct INP1ΔC–BdC–YFP); or (iii) by a single-pass TM domain from a known integral membrane protein, the Arabidopsis receptor-like kinase FER (FER TM; construct INP1ΔC–FER TM–YFP) (Escobar-Restrepo *et al.*, 2007; Liu *et al.*, 2016) (Fig. 5A). Addition of the FER TM region was previously found to be sufficient to tether another near-membrane protein, LORELEI, at the plasma membrane of pollen tubes and synergid cells (Liu *et al.*, 2016).

Also, to test if the addition of a known TM domain to the full-length INP1 could potentially interfere with the INP1 delivery, localization, or formation of the punctate lines (e.g. by immobilizing the protein in the plasma membrane), we added FER TM either between the end of the full-length INP1 and the beginning of YFP (construct INP1–FER TM–YFP) or after the YFP fused to the full-length INP1 (construct INP1–YFP–FER TM) (Fig. 5A). We then evaluated the ability of all these chimeric proteins to restore apertures and form punctate lines in tetrads of the *inp1* mutant.

We found that only two of the five constructs were able to restore apertures and punctate lines (Fig. 5B, B', E, E'). In the first case, the putative TM region of AtINP1 was replaced with the corresponding region from *E. californica* (INP1ΔC–EcC–YFP) (Fig. 5B, B'), demonstrating that the sequence differences between AtINP1 and EcINP1 in this particular region were not responsible for the failure of the full-length EcINP1 to rescue Arabidopsis apertures. The second case, which resulted only in a partial aperture restoration/puncta formation, involved the construct in which the FER TM domain was introduced between the full-length INP1 and YFP (INP1–FER TM–YFP) (Fig. 5E, E'). In this case, apertures, usually shorter than normal, were restored and puncta formed in 65% of the T_1_ plants (*n*=48), suggesting that the addition of FER TM has some negative effect on the efficiency of the INP1 localization/assembly. However, contrary to our hypothesis that the presence of an actual TM domain might immobilize INP1 throughout the plasma membrane, the chimeric protein did not exhibit an obvious plasma membrane accumulation.

The three other chimeric proteins were unable to restore apertures. In the case when FER TM replaced the putative INP1 TM (INP1ΔC–FER TM–YFP), the diffuse YFP signal was present throughout the tetrad-stage microspores but no puncta formed (Fig. 5D'), indicating that this C-terminal region of INP1 is necessary for INP1 localization and assembly into puncta and that a TM domain from an unrelated protein is not sufficient to perform this function.

Interestingly, in the cases when either FER TM was added after the INP1–YFP fusion (INP1–YFP–FER TM) or when the C-terminal region in AtINP1 was replaced by the corresponding region of BdINP1 (INP1ΔC–BdC–YFP), the YFP signal was no longer found in punctate aggregates on the microspore surface but instead formed punctate inclusions inside the microspores (Fig. 5C', F'). This suggests that these particular modifications interfered with the ability of INP1 to get through a sorting pathway successfully to the microspore surface. Together, the results from the expression of these three chimeric constructs indicate that the ability of INP1 to get to the membrane surface and assemble there in punctate lines is an essential prelude for aperture formation.

### Arabidopsis–tomato INP1 chimeras reveal the importance of the central portion of the protein for its localization and function

The fragment fusion approach described earlier led to the apparent destabilization of truncated proteins and did not allow us to identify specific regions required for INP1 localization and function (Fig. 4). Given that the full-length INP1 proteins from species such as tomato are stable in Arabidopsis but unable to form punctate lines and rescue apertures, we reasoned that creating interspecific chimeric proteins between AtINP1 and its homolog from tomato (SlINP1) and testing them in Arabidopsis would be likely to be be a more fruitful approach to finding domains required for INP1 function and localization.

**Fig. 6.**
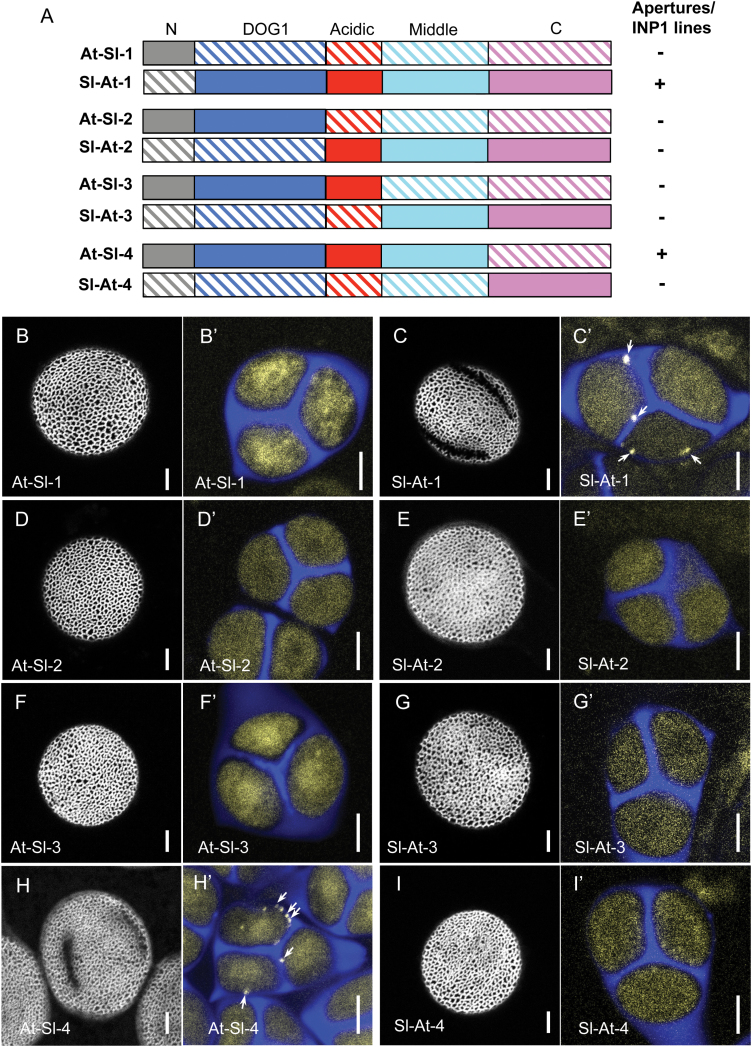
The central portion of INP1 is required for species-specific functionality in Arabidopsis. (A) A diagram of chimeric constructs with portions of INP1s from Arabidopsis and tomato. The domain color scheme is the same as in previous figures. The Arabidopsis sequences are shown as solid colors, and the tomato sequences are indicated by diagonal hatching. (B–I') Pollen aperture phenotypes (B, C, D, etc.) and INP1–YFP expression in tetrads (B', C', D', etc.) in lines expressing different constructs. The callose wall of tetrads is stained with Calcofluor White (blue). Yellow signal indicates the presence of INP1–YFP. While tetrads from all transformants had diffuse INP1–YFP fluorescence, the peripheral puncta and lines of INP1–YFP (arrows) were only visible in tetrads from plants expressing the Sl–At-1 (C') and the At–Sl-4 (H') constructs. Correspondingly, aperture rescue was only observed in the plants expressing Sl–At-1 and At–Sl-4, in which the three central domains came from AtINP1 (C, H). Scale bars=5 μm.

We created a series of eight constructs by replacing one or more of the five regions of AtINP1 shown in Fig. 3 with the corresponding regions from SlINP1 (Fig. 6A): four of the constructs had Arabidopsis sequences at their N-termini and tomato sequences at the C-termini (constructs At–Sl-1 to 4) and, correspondingly, the other four constructs contained tomato sequences at their N-termini and Arabidopsis sequences at their C-termini (constructs Sl–At-1 to 4). All these constructs, driven by *DMC1pr* and containing a *YFP* gene fused to the chimeric *INP1* genes at their C-termini, were then tested in the *inp1* mutant for their ability to restore apertures and form lines at the microspore periphery.

Two of the constructs, Sl–At-1 and At–Sl-4, restored formation of apertures and INP1 assembly into punctate lines (Fig. 6C, C', H, H'). In these constructs, respectively, either the N-terminal domain or the C-terminal domain came from tomato and the remaining four domains from Arabidopsis (Fig. 6A). This suggests that these two domains in AtINP1, while important for stability and function, do not make critical contributions to the species-specific aspects of the protein behavior. We note that aperture restoration was very robust in the presence of the tomato N-terminal domain: all 36 T_1_ plants with the Sl–At-1 construct had long or medium-long apertures restored, indicating efficient complementation. The rescue was somewhat less robust in the presence of the tomato C-terminal domain (At–Sl-4 construct). Although most of the T_1_ plants formed pollen apertures (*n*=9/13), in multiple cases apertures were shorter than normal (Fig. 6H) and about a quarter of plants failed to form apertures altogether, indicating that although the tomato C-terminus was able to substitute for the Arabidopsis domain, this change still had a negative impact on the protein functionality. The corresponding mirror constructs (At–Sl-1 and Sl–At-4), containing most of the protein from tomato fused with either the N- or the C-terminal domain from Arabidopsis, failed to restore formation of apertures and INP1 lines (Fig. 6B, B', I, I'). None of the remaining four constructs (At–Sl-2, Sl–At-2, At–Sl-3, and Sl–At-3), in which the swap affected the three central domains (DOG1, acidic, and the middle), were able to complement aperture defects in Arabidopsis (Fig. 6D–G'). This indicates that these three regions from the central portion of AtINP1 are required for the protein function specifically in Arabidopsis and may be involved in interactions with additional species-specific aperture factors.

## Discussion

### The role of INP1 as an aperture factor appears to be conserved in evolution

Pollen apertures, exhibiting a wide variety of patterns in angiosperms, present an interesting model of cellular and extracellular polarity. In developing pollen of Arabidopsis, formation of apertures involves the generation of distinct membrane domains that become decorated with the INP1 protein. In that species, this novel protein exhibits a highly unusual localization pattern, assembling into three equidistant lines at the microspore periphery. Although many other species have different patterns of apertures, INP1 homologs, encoded in many species by single-copy genes, can be recognized throughout the angiosperms. These homologous proteins, however, show a high sequence divergence, making it difficult to predict whether they, like their Arabidopsis counterpart, are involved in formation of apertures. Furthermore, in our previous study, we have shown that BdINP1, a homolog from the grass Brachypodium, was unable to restore apertures in the Arabidopsis *inp1* mutant (Dobritsa and Coerper, 2012), potentially bringing into question the functional conservation of these proteins.

However, the results presented here suggest that INP1 involvement in pollen aperture formation is conserved in evolution. Just like the *inp1* mutant in Arabidopsis, the maize mutant defective in ZmINP1 loses its apertures. This result is particularly striking, given the dramatic difference between the structures and patterns of apertures in these two species—three equatorial furrows in Arabidopsis versus a single polar pore surrounded by a ring-shaped annulus and covered by a lid-like operculum in maize—as well as the very significant differences in the sequences of the corresponding INP1 proteins, which share only 36% identity. This finding strongly suggests that INP1 was involved in pollen aperture formation prior to the evolutionary split between the monocots and eudicots. Although it remains to be seen whether ZmINP1 localizes to the distal sites on the surfaces of maize microspores where the single pore develops, we expect that this might be the case. In other grasses, which share highly similar aperture patterns and high levels of sequence identity among their INP1 homologs, these proteins are likely to be similarly involved in aperture formation. In addition, the conservation of the INP1 role between Arabidopsis and maize also implies a highly probable conservation of function for INP1s from other eudicots, given that their aperture patterns and their INP1 homologs are much more similar to the Arabidopsis apertures and AtINP1 than the corresponding counterparts from grasses.

### Apertures play a critical role in maize fitness

The loss of male fertility in the *inp1* mutant of maize demonstrates the essential role the single pore in pollen of this species plays in pollen tube emergence. It is likely that pollen tubes of other grasses are similarly dependent on the presence of apertures. Although in many species pollen tubes seem to emerge specifically through the apertures (Heslop-Harrison, 1979; Heslop-Harrison and Heslop-Harrison, 1985; Edlund *et al.*, 2016) and it has long been assumed that pollen tube exit is one of the primary aperture functions (Wodehouse, 1935; Heslop-Harrison, 1968; Edlund *et al.*, 2004), our results provide the most direct evidence for the critical fitness role that these morphological features play in some species. This is in contrast to the aperture loss in Arabidopsis, where *inp1* mutants show no gross fertility defects under laboratory conditions (Dobritsa *et al.*, 2011), as well as to the observations in wild-type Arabidopsis and several other species of Brassicaceae whose pollen tubes exhibit the ability to choose the most direct route to the stigma and frequently break through the exine rather than taking a detour through one of the three apertures (Edlund *et al.*, 2004; Hoedemaekers *et al.*, 2015; Edlund *et al.*, 2016). It is likely that the thicker exine in the pollen of maize and, in particular, the presence of the highly covered tectum, the roof-like layer of exine (Skvarla and Larson, 1966) [annotated in PalDat (www.paldat.org) as eutectate in grasses versus semi-tectate in Arabidopsis and other Brassicaceae] necessitates the strict dependence on apertures for germination in that species.

### Divergent factors besides INP1 are probably involved in formation of apertures

When AtINP1 was replaced with its homologs from other eudicots and the monocot Brachypodium, closely related proteins were able to localize and function properly in Arabidopsis, whereas the more distant homologs failed at this. These interspecies complementation experiments allowed us to draw several important conclusions. (i) Given the conserved role of INP1 as an aperture factor in such distant species as Arabidopsis and maize, the inability of several INP1 orthologs to function in Arabidopsis suggests that in their respective species they rely on interactions with co-evolved partners, which, in the cases of tomato, poppy, and Brachypodium, must have significantly diverged from their Arabidopsis counterparts. (ii) Importantly, the fact that MiINP1 was able to rescue apertures indicates that the *INP1* gene in Matthiola is functional. It is also expressed in the same organs and at the same developmental stages as the Arabidopsis INP1, since the *MiINP1* cDNA was isolated from the developing anthers. In turn, the ability of MiINP1 to function allows us to postulate the existence of at least one additional gene required for aperture formation, mutations in which could explain the absence of apertures in Matthiola. (iii) The fact that the apertures restored in the presence of CrINP1 looked like the Arabidopsis apertures and not like those of Capsella demonstrates that INP1 is not responsible for all aspects of aperture morphology.

Taken together, these results indicate that, while INP1 is absolutely required for aperture formation, there must be additional factors that specify particular aspects of aperture morphology. Combining these data with our previous results that INP1 levels do not appear to play a major role in specifying aperture numbers (Reeder *et al.*, 2016; Dobritsa *et al.*, 2017), we propose that assembly of INP1 into peripheral puncta and lines is downstream of the formation of distinct membrane domains, whose number, positions, and some aspects of morphology depend on additional molecular players. Based on the data presented here, at least some of these molecular players are expected to exhibit sequence-specific differences that would allow them to interact with divergent INP1s.

### Ability of INP1 variants to assemble into punctate lines at the cell periphery perfectly correlates with formation of apertures

The experiments involving domain swaps, expression of homologs from other species, and Arabidopsis–tomato chimeras underscored the importance of INP1 punctate lines, as these structures exhibited perfect correlation with pollen aperture formation. No apertures developed when INP1 was expressed but failed to get to the plasma membrane and assemble into punctate lines. We have not observed a situation where INP1 lines formed but apertures did not develop or vice versa. While we have previously assumed that the INP1 ability to form punctate lines is necessary for aperture formation (Dobritsa and Coerper, 2012), the results presented here provide strong support for this assumption.

### Structure–function analysis of INP1 suggests that the central portion of the protein is particularly important for species-specific interactions

Because INP1 has no domains of known function and tends to get destabilized when its fragments are used, the question of which of its portions are required for its delivery and assembly at specific membrane positions is especially challenging. The use of Arabidopsis–tomato INP1 chimeras suggested that the three central domains of INP1 are involved in interactions with species-specific factors that help INP1 to localize and perform its function.

Two of these regions, the acidic domain and the middle domain, are quite divergent in different species, emphasizing the possibility that they contain amino acids critical for species-specific interactions between INP1 and other aperture factors. Within the acidic domains in AtINP1 and in the other two Brassicaceae INP1s used in this study (CrINP1 and MiINP1, both functional in Arabidopsis), about a third of the sequence is represented by aspartate or glutamate residues (13/40 in AtINP1). In contrast, in the INP1s from tomato and California poppy, which failed to function in Arabidopsis, aspartate or glutamate occupy only about a sixth of the corresponding sequence (6/35 in SlINP1 and 6/36 in EcINP1). Further studies will be required to understand whether the functionality of this region is determined by specific amino acids or by a net charge. The third domain of the central region, DOG1, is significantly more conserved in eudicots (AtINP1 and SlINP1 have 56% identity in this domain, compared with 31% and 38% identity in the other two domains), so it was somewhat surprising that the tomato DOG1 domain was not functional in Arabidopsis. Although the function of the plant-specific DOG1 domain is presently unknown, it has been hypothesized to be involved in protein interactions (Magnani *et al.*, 2014).

The C-terminal domain of INP1 has drawn our attention because in multiple species it was predicted to contain a possible TM domain (Dobritsa and Coerper, 2012). The replacement of this region with a corresponding sequence from tomato resulted in a protein that was able to localize and function correctly, albeit with lower efficiency than the normal AtINP1. Similarly, a replacement of the subportion of this domain, corresponding to the putative TM domain, with the sequence from the California poppy homolog EcINP1 was sufficient to restore apertures. Yet, the replacement of this region either with the Brachypodium sequence or with a *bona fide* TM domain did not create a functional protein, potentially casting doubt on the idea that INP1 contains a true TM domain.

Similar to the C-terminal domain, the much more divergent N-terminal domain (37% identity between AtINP1 and SlINP1) did not appear to provide species specificity. In many species, including Arabidopsis and tomato, this domain has several arginine and lysine residues and meets the criteria for containing a basic and hydrophobic (BH) motif (Brzeska *et al.*, 2010). As such motifs can participate in charge-related interactions with membrane phospholipids (Bailey and Prehoda, 2015; Barbosa *et al.*, 2016; Simon *et al.*, 2016), this region of INP1 may potentially be involved in keeping the protein at the specific membrane domains.

In summary, we demonstrated that the involvement of INP1 in aperture formation is conserved in very different species, despite the significant divergence of protein sequences and aperture patterns. Our data further suggested that the INP1 role as an aperture factor probably depends on the presence of additional species-specific factors and that the central region of INP1 is particularly important for species-specific interactions. Identification of other aperture factors and of specific subregions of INP1 involved in interactions with these factors will require further investigation.

## Supplementary data

Supplementary data are available at *JXB* online.

Table S1. Primers used in this study.

Fig. S1. INP1 is non-functional in the presence of an N-terminal tag.

## Supplementary Material

Supplementary Figure S1 and Table S1

## Abbreviations:

DMC1pr: *DMC1* promoter
FER: FERONIA
INP1: INAPERTURATE POLLEN 1
TM: transmembrane
YFP: yellow fluorescent protein.
