# Supplementary material for: INP1 involvement in pollen aperture formation is evolutionarily conserved and may require species-specific partners
Source: J Exp Bot. 2017 Nov 28;69(5):983–96. doi: 10.1093/jxb/erx407 (PMC5965098; doi:10.1093/jxb/erx407)
Supplement: Supplementary Figure S1 and Table S1 [file erx407_suppl_supplementary_figure_s1_table_s1.pdf]

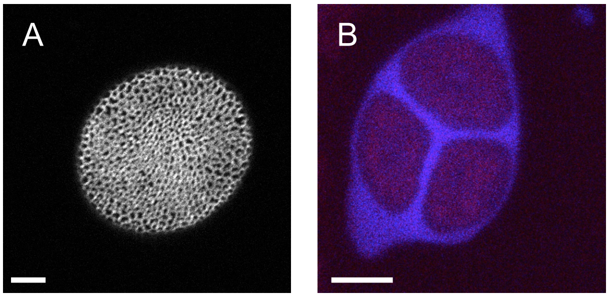

Supplementary Fig. S1. **INP1 is non-functional in the presence of an N-terminal tag.** (A) No pollen apertures are restored and (B) no INP1 puncta are formed in the presence of the *DMC1pr:mRuby2-INP1* construct. In (B) callose wall is blue and mRuby2 signal is red. Scale bars – 5 μm.

Supplementary Table S1. Primers used in this study.

Primer	Primer sequence (5' to 3')	Enzyme or In-Fusion (IF)	Used for:
ZmINP1-BF	GTCTCAGCCCACCGCTGTATCCATC		Genotyping maize (used to amplify the WT band in ZmINP1)
ZmINP1-BR	GTCGAAGCGGCGGAGCACCTC		Genotyping maize (used to amplify the WT band in ZmINP1 and the mutant band in *zminp1*)
TIR6	AGAGAAGCCAACGCCAWCGCCTCYATTTCGTC		Genotyping maize (used to amplify the mutant band in *zminp1*)
AgeI-INP1-EF	ggaaaccggtATGCCTTTCTCTTTCTTCTCC	*Age*I	*DMC1pr:INP1-YFP*; N-terminal fragment constructs, *DMC1pr:CrINP1-YFP*
NcoI-INP1-FR	ggaaccatggcATTGGGCAAAGAAAGAATCTC	*Nco*I	*DMC1pr:INP1-YFP*; C-terminal fragment constructs
SacI-DMCIpr-F	ggaagagctcTAAAATTAATTTGATTAGTGGATC	*Sac*I	*DMC1pr:INP1-YFP* (*DMC1* promoter)
AgeI-DMC1pr-R	ggaaaccggtTTTCTCGCTCTAAGAGTCTCTAAGC	*Age*I	*DMC1pr:INP1-YFP* (*DMC1* promoter)
INP1-L6-R-NcoI	ggaaccatggcAAAATCTTGAAGAAGAATCTG	*Nco*I	INP1 N-fragment construct (INP1$_{1\text{-}265}$)
INP1-KR6-NcoI	ggaaccatggcAAACCCAGCAAGAAACTGAC	*Nco*I	INP1 N-fragment construct (INP1$_{1\text{-}258}$)
INP1-KR5-NcoI	ggaaccatggcAGCCGCTTGATGCTCACTTGT	*Nco*I	INP1 N-fragment construct (INP1$_{1\text{-}244}$)
INP1-L7-R-NcoI	ggaaccatggcACTCTTCCTCAAACGATTCGCATC	*Nco*I	INP1 N-fragment construct (INP1$_{1\text{-}230}$)
INP1-KR2-NcoI	ggaaccatggcAAGCTTCTTCTTCTTCCCAAC	*Nco*I	INP1 N-fragment construct (INP1$_{1\text{-}194}$)
INP1-L8-R-NcoI	ggaaccatggcCTTCCCAACTTGATACGACG	*Nco*I	INP1 N-fragment construct (INP1$_{1\text{-}190}$)
INP1-KR1-NcoI	ggaaccatggcGATGAAAGAACGGAGGAGATTGGTGAG	*Nco*I	INP1 N-fragment construct (INP1$_{1\text{-}109}$)
INP1-L1-	ggaaaccggtATGTTCAATGAT	*Age*I	INP1 C-fragment construct (INP1$_{15\text{-}273}$)

F-AgeI	TTCTACGAAGACTGG		
INP1-L2-F-AgeI	ggaaaccggtATGCTAACCGAAAACTGCCTCCCTCT	*Age*I	INP1 C-fragment construct (INP1$_{26-273}$)
INP1-L3-F-AgeI	ggaaaccggtATGTCCGCTTCCGTCCTCTCCTCCAACG	*Age*I	INP1 C-fragment construct (INP1$_{43-273}$)
INP1-L4-F-AgeI	ggaaaccggtATGCCTTACCTTCTCTTCCCTTCATGG	*Age*I	INP1 C-fragment construct (INP1$_{75-273}$)
INP1-KF5-AgeI	ggaaaccggtATGTTTCTTTTCCTCGGCGATATC	*Age*I	INP1 C-fragment construct (INP1$_{90-273}$)
INP1-L5-F-AgeI	ggaaaccggtATGCAACCGTTAAAGATGACGATGG	*Age*I	INP1 C-fragment construct (INP1$_{131-273}$)
NcoI-CrINP1-2R	ggaaccatggcATTGGGCAATGCAAGAATC	*Nco*I	*CrINP1*
AgeI-Min-EF	ggaaaccggtATGCCTTTCTCTCTCTTCTCC	*Age*I	*MiINP1*
NcoI-MiINP1-14R	ggaaccatggcGTTAGGCAATGAAAGGAGC	*Nco*I	*MiINP1*
SlyINP1-F-AgeI	ggaaaccggtATGTTCAAAGCCATAGCTCATTTGGGTTCAAGAAATCTTCG	*Age*I	*SlINP1*
SlyINP1-R-NcoI	ggaaccatggcTAATTCCAAAGAACACTTGTCAAATTGAC	*Nco*I	*SlINP1*
EcaINP1-F-AgeI	ggaaaccggtATGATCAAAGCTGCAGCTCGA	*Age*I	*EcINP1*
EcaINP1-R-NcoI	ggaaccatggcAATGCCTGATAATGGAATCTTGC	*Nco*I	*EcINP1*
DMC1pr-INP1-IF-F	GAGCGAGAAAaccggtATGCCTTTCTCTTTCTTCTCCCG	IF	*INP1-FER TM-YFP*; *INP1ΔC-FER TM-YFP*; *INP1-YFP-FER TM; INP1ΔC-EcC-YFP; INP1ΔC-BdC-YFP; At-Sl-1 to 4*
INP1-FER TM-IF-R	TTGCTTTTATTGGGCAAAGAAAGAATCTCAAAATCTTGAAG	IF	*INP1-FER TM-YFP*
INP1-FER TM-IF-F	GCCCAATAAAAGCAATACGGCTATTATTGCAGGC	IF	*INP1-FER TM-YFP*
FER TM-	cccttgctcaccatggcGTAATCAC	IF	*INP1-FER TM-YFP; INP1ΔC-FER TM-YFP*

YFP-IF-R	CACGCTTACGTCTGC		
INP1dTM-FTM-IF-R	TTGCTTTTGTCCATAATCACACTCTTCCTCAAACGA	IF	*INP1ΔC-FER TM-YFP*
INP1dTM-FTM-IF-F	TATGGACAAAAGCAATACGGCTATTATTGCAGG	IF	*INP1ΔC-FER TM-YFP*
YFP-FTM-IF-R	TTGCTTTTcttgtacagctcgtccatgcc	IF	*INP1-YFP-FER TM*
YFP-FTM-IF-F	gtacaagAAAAGCAATACGGCTATTATTGCAGGC	IF	*INP1-YFP-FER TM*
FTM-pGR111-IF-R	agcgtaccggaCTAGTTCAGTAATCACCACGCTTACGTCTGC	IF	*INP1-YFP-FER TM*
INP1dTM-EcTM-IF-R	GTTAAAACACTCTTCCTCAAACGATTCGC	IF	*INP1ΔC-EcC-YFP*
EcTM-IF-F	GAAGAGTGTTTTAACAGAGATTATCGGTGCTACC	IF	*INP1ΔC-EcC-YFP*
EcTM-YFP-IF-R	cccttgctcaccatggcAATGCCTGATAATGGAATCTTGCATTGT	IF	*INP1ΔC-EcC-YFP*
INP1dTM-BdTM-IF-R	AGAACGCCCTTCCTCAAACGATTCGCATCAACG	IF	*INP1ΔC-BdC-YFP*
BdTM-IF-F	GAGGAAGGGCGTTCTTTCGGAGCTCG	IF	*INP1ΔC-BdC-YFP*
BdTM-YFP-IF-R	cccttgctcaccatggcACTACCAGGCGAGGCG	IF	*INP1ΔC-BdC-YFP*
Min-4F	ATGCCTTTCTCTTTCTTCTC		Isolation of MiINP1
Min-5R	TCCATGATCACACTCTTCCTC		Isolation of MiINP1
1-At-Sl-IF-R	ACGGAGCTGAGGGAGGCAGTTTTCGGTTAGGGTTTTTGAC	IF	*At-Sl-1*
1-Sl-F	CTCCCTCAGCTCCGTCAC		*At-Sl-1*
SlINP1-	cccttgctcaccatggcTAATTCCA	IF	*At-Sl-1 to 4*

YFP-IF-R	AAGAACACTTGTCAAATT G GAGCGAGAAAaccggtATGT TCAAAGCCATAGCTCAT		
DMC1pr-Sl-INP1-IF-F	GAGCGAGAAAaccggtATGT TCAAAGCCATAGCTCAT	IF	*Sl-At-1 to 4*
1-Sl-At-IF-R	ACGGAGGAGAGGGAGGA GAACGTTCTTGAGAGTCT TG	IF	*Sl-At-1*
1-At-F	CTCCCTCTCCTCCGTCAAT C		*Sl-At-1*
AtINP1-YFP-IF-R	cccttgctcaccatggcATTGGGCA AAGAAAGAATCTC	IF	*Sl-At-1 to 4*
2-At-Sl-IF-R	GGATTCTGAGTCGCCGAT GAAAGAACGGAGGAGATT G	IF	*At-Sl-2*
2-Sl-F	GGCGACTCAGAATCCGAA ATTGAC		*At-Sl-2*
2-Sl-At-IF-R	TTGATTCTCACGGTCGATA AATGAACGGAGGAGATTT G	IF	*Sl-At-2*
2-At-F	GACCGTGAGAATCAAGAT TCCGAC		*Sl-At-2*
3-At-Sl-IF-R	GCACTCGATTTGATCGATT CTTTTAACCAACTCATCAG	IF	*At-Sl-3*
3-Sl-F	GATCAAATCGAGTGCGGA TTGAG		*At-Sl-3*
3-Sl-At-IF-R	ACACTCAATCTGATCGAC CCCAGTAGTTAATTTCCTC G	IF	*Sl-At-3*
3-At-F	GATCAGATTGAGTGTACA ATGAGAC		*Sl-At-3*
4-At-Sl-IF-R	CAGTTCTTCCATCTCAATC TTTGCTGCTTCATCCAC	IF	*At-Sl-4*
4-Sl-F	GAGATGGAAGAACTGGTC GG		*At-Sl-4*

4-Sl-At-IF-R	AAGCTCTTCCATCTCCGCCGCCGATGATTCTCC	IF	*Sl-At-4*
4-At-F	GAGATGGAAGAGCTTGTGAG		*Sl-At-4*
DMC1pr-mRuby2-IF-F2	gagcgagaaaaccggATGGTGTCTAAGGGCGAAGAGCTG	IF	*DMC1pr:mRuby2-INP1*
mRuby2-INP1-IF-R	AGAGAAAGGaccggtCTTGTACAGCTCGTCCATCCCAC	IF	*DMC1pr:mRuby2-INP1*
INP1-IF-F	accggtCCTTTCTCTTTCTTCTC	IF	*DMC1pr:mRuby2-INP1*
INP1-pGR111-Nco-IF-R	CCCTTGCTCACCATGGTTAGAATTTGTTTTCCTCTCACTC	IF	*DMC1pr:mRuby2-INP1*
